# Histone Chaperone Nrp1 Mutation Affects the Acetylation of H3K56 in *Tetrahymena thermophila*

**DOI:** 10.3390/cells11030408

**Published:** 2022-01-25

**Authors:** Yinjie Lian, Huijuan Hao, Jing Xu, Tao Bo, Wei Wang

**Affiliations:** 1 Key Laboratory of Chemical Biology and Molecular Engineering of Ministry of Education, Institute of Biotechnology, Shanxi University, Taiyuan 030006, China; 201613002003@email.sxu.edu.cn (Y.L.); 201813002002@email.sxu.edu.cn (H.H.); xujing@sxu.edu.cn (J.X.); botao@sxu.edu.cn (T.B.); 2 College of Life Sciences, Shanxi University, Taiyuan 030006, China

**Keywords:** *Tetrahymena thermophila*, histone chaperone *NRP1*, mutation, acetylation of H3K56, genome transcription

## Abstract

Histone modification and nucleosome assembly are mainly regulated by various histone-modifying enzymes and chaperones. The roles of histone-modification enzymes have been well analyzed, but the molecular mechanism of histone chaperones in histone modification and nucleosome assembly is incompletely understood. We previously found that the histone chaperone Nrp1 is localized in the micronucleus (MIC) and the macronucleus (MAC) and involved in the chromatin stability and nuclear division of *Tetrahymena thermophila*. In the present work, we found that truncated C-terminal mutant HA-Nrp1^TrC^ abnormally localizes in the cytoplasm. The truncated-signal-peptide mutants HA-Nrp1^TrNLS1^ and HA-Nrp1^TrNLS2^ are localized in the MIC and MAC. Overexpression of Nrp1^TrNLS1^ inhibited cellular proliferation and disrupted micronuclear mitosis during the vegetative growth stage. During sexual development, Nrp1^TrNLS1^ overexpression led to abnormal bouquet structures and meiosis arrest. Furthermore, Histone H3 was not transported into the nucleus; instead, it formed an abnormal speckled cytoplastic distribution in the Nrp1^TrNLS1^ mutants. The acetylation level of H3K56 in the mutants also decreased, leading to significant changes in the transcription of the genome of the Nrp1^TrNLS1^ mutants. The histone chaperone Nrp1 regulates the H3 nuclear import and acetylation modification of H3K56 and affects chromatin stability and genome transcription in *Tetrahymena*.

## 1. Introduction

The genome of the eukaryotic cell is packaged into chromatin. Thus, dynamic changes in chromatin structure and composition could potentially affect the function of genomes. The nucleosome is the fundamental building block of chromatin. The correct assembly and disassembly of nucleosomes are imperative for genome transcription, replication, and repair [1]. Histones H3 and H4 form the core of the nucleosome, while histones H2A and H2B bind at the periphery of the nucleosome [2]. Histone chaperones regulate histone assembly and disassembly [3], promote the interaction of new histones with other chaperones [4], mediate histone epigenetic modification [5], participate in ATP-dependent nucleosome remodeling [6], and regulate histone exchange during transcription [7]. These chaperones also provide the functional complexity of nucleosomes by incorporating histone variants and combinatorial post-translational modifications (PTMs), which precisely regulate gene expression and the nuclear architecture. In HeLa cells, canonical histone H3 is deposited into the genome with the assistance of the histone chaperone CAF-1 complexes via replication-coupled nucleosome assembly (RC). The histone variant H3.3 is deposited by the histone chaperones HIRA and Daxx via replication-independent (RI) nucleosome assembly [8,9]. The two pathways (RC and RI) contain three common histone chaperones, namely nuclear autoantigenic sperm protein (NASP), the small subunit of the CAF-1 complex (Caf1c), and anti-silencing factor (Asf1) [8]. NASP and Caf1c directly bind to H3 and H4 and integrate histone acetyltransferase 1 (HAT1) to mediate conserved H4K5 and K12 acetylation before positioning [10,11]. DNA-damage sensitivity correlates with decreases in the acetylation level of H3K56 in yeast [12,13]. Incorporation of histone H3K56ac into the nucleosome is essential for chromatin remodeling, DNA replication, and repair [14]. In mammalian cells, H3K56 acetylation is catalyzed by different acetyltransferases, including CBP, p300, and Gcn5 [15,16]. CBP and P300 acetylate multiple substrates and preferentially catalyze the acetylation of H3 N-terminal lysines [15,17]. Gcn5 and five lysine residues of the H3 N terminus are required for efficient deposition of new H3 onto replicating DNA [18,19]. H3K56ac primarily localizes in active chromatin regions and is involved in cancer progression, DNA double-strand break repair, genome stability maintenance, and cell pluripotency [20]. 

*Tetrahymena thermophila* is an excellent model organism for studies on histone modification, owing to its unique nuclear dimorphism, which segregates the somatic macronucleus (MAC) and the germline micronucleus (MIC) in the same cytoplasm; the MAC is transcriptionally active, whereas the MIC is transcriptionally silent in the vegetative growing stage [21]. MAC and MIC exhibit different chromatin structures and asynchronous division [22]. During vegetative growth, the MAC divides amitotically, while the MIC divides mitotically [23]. After the cells are starved for 24 h, conjugation is triggered when *Tetrahymena* cells of two different mating types are mixed together. The parental MIC undergoes meiosis, four haploid gamete nuclei are formed, and one of them is selected. The selected nucleus undergoes mitosis, and reciprocal fertilization gives rise to a diploid zygotic nucleus in each cell. The zygotic nucleus undergoes two mitoses and two new MICs, and new MACs are formed [24]. The parental MAC was destroyed through autophagy-mediated programmed nuclear death [25]. The pair members separate, forming ex-conjugants in which one of the two MICs is resorbed. The entire sexual reproduction needs ~24 h [21,24]. Modification of H3K56 acetylation occurs extensively in MAC. However, Hat1 and Hat2/Gcn5 fail to acetylate H3K56, and Asf1 is not required to acetylate H3K56 in *T. thermophila* [26]. It was found recently by us that Nrp1 is required for chromatin stability and nuclear division [27]. However, the epigenetic effect of Nrp1 remains poorly understood. In the present work, we found that Nrp1 mutants inhibit cellular proliferation and lead to abnormal MIC mitosis and MAC amitosis during the vegetative growth stage. *NRP1* mutations affected the acetylation level of H3K56 and H3 nuclear import. During the conjugation stage, the Nrp1^TrNLS1^ mutant inhibited MIC elongation and the progress of meiosis. Furthermore, genome transcription was significantly modified in the Nrp1 mutants. *NRP1* mutation affects H3K56 acetylation, H3 nuclear import, and genome transcription in *Tetrahymena.*

## 2. Materials and Methods

### 2.1. Strains and Culture

Different mating types of *T. thermophila* strains, including B2086 (II), CU427 (VI), and CU428 (VII), were originally obtained from the National *Tetrahymena* Stock Center (http://tetrahymena.vet.cornell.edu/, accessed on 8 July 2021, Cornell University, Ithaca, NY, USA). The cells were cultured in 1× SPP (Super Proteose Peptone) medium at 30 °C [28]. The log-phase growing cells were starved in 10 mM Tris (pH 7.4) at 30 °C, without shaking, for 24 h [29]. Sexual reproduction was triggered by mixing equal numbers (~2 × 10^5^ cells/mL) of starved cells from two different mating types.

### 2.2. Protein HA Tagging

To create the *NRP1* overexpression plasmid, *NRP1* (TTHERM_01014770) was PCR amplified using primers OE-*NRP1*-F/OE-*NRP1*-R (Table S1). The truncated mutants, including Nrp1^TrNLS1^ (NLS1 deletion, 502–510), Nrp1^TrNLS2^ (NLS2 deletion, 456–463), and Nrp1^TrC^ (C-terminal deletion, 444–510), were amplified from the genomic DNA by PCR, using primers OE-*NRP1*^TrNLS1^-F/OE-*NRP1*^TrNLS1^-R, OE-*NRP1*^TrNLS2^-F1 /OE-*NRP1*^TrNLS2^-R1, OE-*NRP1*^TrNLS1^-F2/OE-*NRP1*^TrNLS1^-R2, OE-*NRP1*^TrC^-F/OE-*NRP1*^TrC^-R (Table S1), respectively. Detailed plasmid construction and transformation steps were performed as previously described [27]. The mutants were confirmed by PCR amplification, using the primers OE-J-*NRP1*-F/OE-J-*NRP1*-R (Supplementary Table S1). 

### 2.3. RNA Extraction and qRT-PCR

First, 8 × 10^5^ cells were collected, and total RNA was extracted by using Trizol reagent (Takara Biotechnology, Dalian, China). RNA samples were pretreated with gDNA Eraser at 42 °C for 5 min, and then cDNA was synthesized by using the PrimerScriptTM RT reagent kit (Takara Biotechnology, Dalian, China). Gene transcription level was tested by qRT-PCR. RT-*NRP1*-F/RT-*NRP1*-R primer pairs, 17s-F/17s-R primer pairs (Supplementary Table S1), and SYBR Green II mix (SYBR^®^Premix Ex Taq™ Kit, Takara Biotechnology, Dalian, China) were used in this experiment, with 17S rRNA as the internal control. The PCR program was as follows: 30 s at 95 °C, 40 cycles of 5 s at 95 °C, and an extension for 2 min at 60 °C. 

### 2.4. Indirect Immunofluorescence

First, 6 × 10^5^ cells were collected and fixed overnight at 4 °C in Lavdowsky’s fixative (ethanol:formalin:acetic acid:water, 50:10:1:39) for HA immunofluorescence stain or in Schaudinn’s fixative (2:1, saturated HgCl_2_:100% ethanol) for α-tubulin immunofluorescence stain. These samples were blocked with blocking solution for 1 h. Then samples were incubated overnight with mouse anti-HA antibody (1:500 dilution; Clone 114-2C-7, Millipore, Burlington, MA, USA) or anti-α-tubulin antibody (1:200 dilution; T6074, Sigma, Santa Clara, CA, USA) at 4 °C, and then the samples were incubated with TRITC-conjugated anti-mouse IgG antibody (1:800 dilution; AP192R, Millipore, Billerica, MA, USA) for 1 h, at RT. Finally, the samples were stained with 1 μg/mL DAPI for 15 min at RT, and observed by a Delta Vision Elite deconvolution microscope system (Applied Precision/GE Healthcare).

For immunofluorescence analysis of H3 and H3K56ac, 3 mL of the cell cultures was collected, and the cells were fixed in 4% paraformaldehyde for 30 min [20]. The cells were washed in PBS, permeabilized in 0.05% Triton X-100 for 15 min, and then blocked in PBS for 1 h at RT. The cells were incubated overnight with rabbit antiserum anti-H3 (1:500 dilution; #4499, CST, Danvers, MA, USA) and anti-H3K56ac (1:500 dilution; AB_2661786, Active Motif, Carlsbad, CA, USA) at 4 °C. After extensive washing in PBS, the cells were incubated in fluorescein isothiocyanate (FITC)-conjugated anti-rabbit immunoglobulin G (1:1000 dilution; ZF-0312, ZSGB, Beijing, China) diluted 1:1000 for 1 h at RT. The samples stained with 1 μg/mL DAPI for 15 min at RT.

### 2.5. Histone Isolation

Histones were extracted from *Tetrahymena* samples by using the EpiQuik™ Total Histone Extraction Kit (EPIGENTEK, Farmingdale, NK, USA). Cells at a density of 1 × 10^7^ cells/mL were suspended in diluted 1× pre-lysis buffer and then lysed on ice for 10 min, with gentle stirring. The cells were then centrifuged for 1 min at 10,000 rpm and 4 °C. The supernatant was removed, and the cell pellet was resuspended in 3 volumes of lysis buffer, incubated on ice for 30 min, and then centrifuged for 5 min at 12,000 rpm and 4 °C. The supernatant was transferred into a new vial and immediately added with 0.3 volume of Balance-DTT Buffer. Finally, the extract was aliquoted and stored at −80 °C.

### 2.6. Western Blotting

First, 1 × 10^7^ cells were collected. The pellet was dissolved in 1× SDS sample buffer and then boiled for 6 min. The soluble protein samples were separated by 12% SDS/PAGE and transferred to PVDF (polyvinylidene difluoride membranes). The membrane was blocked with 5% skim milk in TBST (100 mM Tris-HCl (pH 7.4), 0.9% NaCl, and 0.05% Tween-20) for 1 h at RT, and incubated with the antibodies, mouse anti-HA antibody (1:500 dilution; Clone 114–2C-7, Millipore, Billerica, MA, USA), or mouse anti-α-tubulin antibody (1:1000 dilution; T9026, Sigma, Santa Clara, CA, USA), HRP-conjugated secondary antibody (1:1000 dilution; A4416, Sigma, Santa Clara, CA, USA). Visualization was achieved with a SuperSignal chemiluminescence detection system.

Extracted histone samples was separated by 15% SDS–PAGE gels and transferred to PVDF. The membrane was blocked with 5% skim milk in TBST and incubated with the antibodies H3K4me3 (1:500 dilution; ab8580, abcam, Cambridge, UK), H4K5ac (1:300; bs-10721R, Bioss, Beijing, China), and H3K56ac (1:500; AB_2661786, USA) and HRP-conjugated secondary antibody (diluted 1:1000, A9169, Sigma, Santa Clara, CA, USA). Visualization was achieved with a SuperSignal chemiluminescence detection system.

### 2.7. Micronuclear Integrity Assay

Micronuclear chromosomes contain micronuleus-specific chromosome breakage sequences (Cbs). The integrity of five micronuclear chromosomes was analyzed by using 10 pairs of specific primers flanking Cbs by PCR [30], and JMJ1 was used as the internal control. Ten pairs of Cbs primer pairs and JMJ1-F/JMJ1-R primer pairs were used in this experiment (Supplementary Table S1). The PCR program was as follows: initial denaturation at 5 min at 94 °C, 32 cycles of 30 s at 94 °C, 30 s at 56 °C, 1 min at 68 °C, and 5 min at 68 °C. 

### 2.8. Flow Cytometry

Cells were prepared as previously described, and apoptosis was examined by using flow cytometry [31]. Briefly, the collected cells were washed twice with pre-cooled PBS and then collected by centrifugation at 3500 rpm for 5 min at 4 °C. The cells were then resuspended in 1× binding buffer and adjusted to a concentration of 1 × 10^6^ cells/mL. Exactly 100 μL of the cell suspension was placed into a 1 mL flow tube and added with 5 μL of Annexin V-PE and 10 μL of 7-AAD (Annexin V-PE/7-AAD Apoptosis Detection Kit, Yeasen Biotechnology, Shanghai). The contents of the tube were mixed and incubated at RT for 15 min in the dark. Exactly 400 μL of 1× binding buffer was added to the tube, the contents were mixed well, and samples were obtained and analyzed by a Guava PCA System flow cytometer (Millipore, Hayward, CA, USA) with a 488 nm argon ion laser light source. A total of 10,000 cells were obtained and examined per sample. Data were analyzed by using GuavaSoft 3.1.1 software, which was provided with the instrument.

### 2.9. RNA Sequencing and Bioinformatic Analysis

First, 1 × 10^6^ cells of each sample were collected. Three sets of biological replicates for wild-type and Nrp1^TrNLS1^ mutant cells, respectively. RNA-Seq analysis of WT or Nrp1^TrNLS1^ mutant cells was performed by Sangon Biotech (Shanghai, China). The total RNA was sequenced by using the Illumina HiSeq XTen platform (Illumina, San Diego, CA, USA). DEGs (differentially expressed genes) were identified according to the following rules: a log2 fold change (FC) ≥ 2, a *p*-value and false discovery rate (FDR) cutoff < 0.1, genes with *p* < 0.05. Furthermore, TopGO (version 2.24.0) was used for GO (Gene Ontology) enrichment analysis (http://www.geneontology.org/, accessed on 8 July 2021), the basic unit of GO is GO-term, and GO enrichment analysis provides all GO terms that are significantly enriched in DEGs compared to the genome background. We also performed a KEGG (http://www.kegg.jp, accessed on 8 July 2021) and KOG enrichment analyses (https://www.ncbi.nlm.nih.gov, accessed on 8 July 2021) by using clusterProfiler. For all analyses, the enrichment threshold was *p* < 0.05.

## 3. Results

### 3.1. Characterization of Nrp1 Nuclear Localization Signals

**Figure 1:**
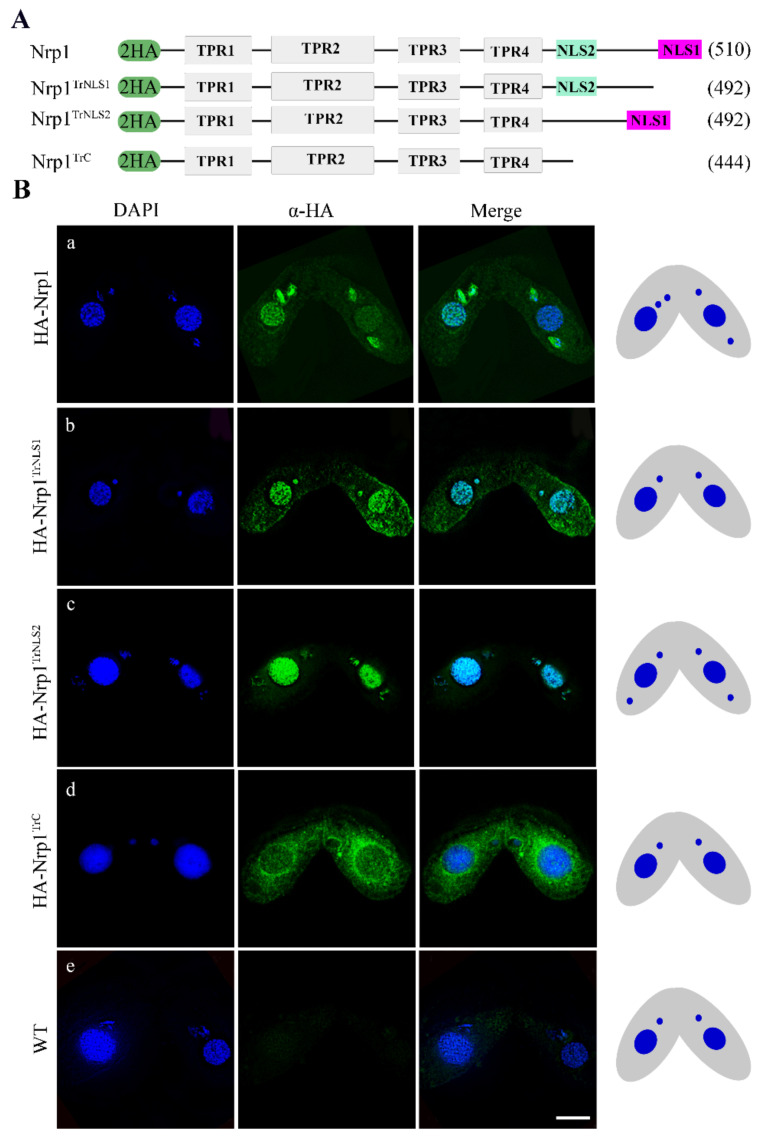
Localization of Nrp1 mutants. (**A**) Schematic representation of Nrp1, Nrp1^TrNLs1^, Nrp1^TrNLs2^, and Nrp1^Trc^ truncated mutants. (**B**) Localization of Nrp1 and Nrp1 mutants during conjugation stage: (**a**) HA-Nrp1, (**b**) HA-Nrp1^TrNLS1^, (**c**) HA-Nrp1^TrNLS2^, and (**d**) HA-Nrp1^TrC^; (**e**) WT cell is as negative control.

The histone chaperone NASP is widely distributed in eukaryotes. The distinctive feature of NASP family proteins is a conserved arrangement of four TPR motifs and an overall negatively charged amino acid sequence [32]. Although NASP family proteins have low similarity in terms of amino acid sequence, they are highly conserved in terms of spatial structure. The *Tetrahymena* genome contains a single NASP homologous gene *NRP1*. The C-terminal region of Nrp1 contains nuclear localization signals (NLS), namely, NLS1 and NLS2 (Supplementary Figure S1A). NLS1 and NLS2 are adjacent in space (Supplementary Figure S1B). Different mutants, including Nrp1^TrNLS1^ (NLS1 deletion, 502–510), Nrp1^TrNLS2^ (NLS2 deletion, 456–463), and Nrp1^TrC^ (C-terminal deletion, 444–510), were created to explore the function of the NLSs. Two hemagglutinin (HA) tags were added to the N-terminus of these fragments, and the mutants were generated by replacing the *MTT1* gene, Cd^2+^ induced overexpression of these fragments (Figure 1A and Supplementary Figure S1C(a–d)). The expression level of the proteins was detected by Western blotting after cells were induced by Cd^2+^ for 12 h (Supplementary Figure S1D). HA-Nrp1 was localized in MAC and MIC during meiotic stage (Figure 1B(a)); HA-Nrp1^TrNLS1^ and HA-Nrp1^TrNLS2^ were also localized in MAC and MIC (Figure 1B(b,c)). By contrast, Nrp1^TrC^ failed to localize in the nucleus and was enriched around the periphery of this organelle (Figure 1B(d)). These results indicate the NLS1 and NLS2 are functionally complementary and determine the success of Nrp1 nuclear import.

### 3.2. Nrp1 Mutation Affects Chromatin Stability

**Figure 2:**
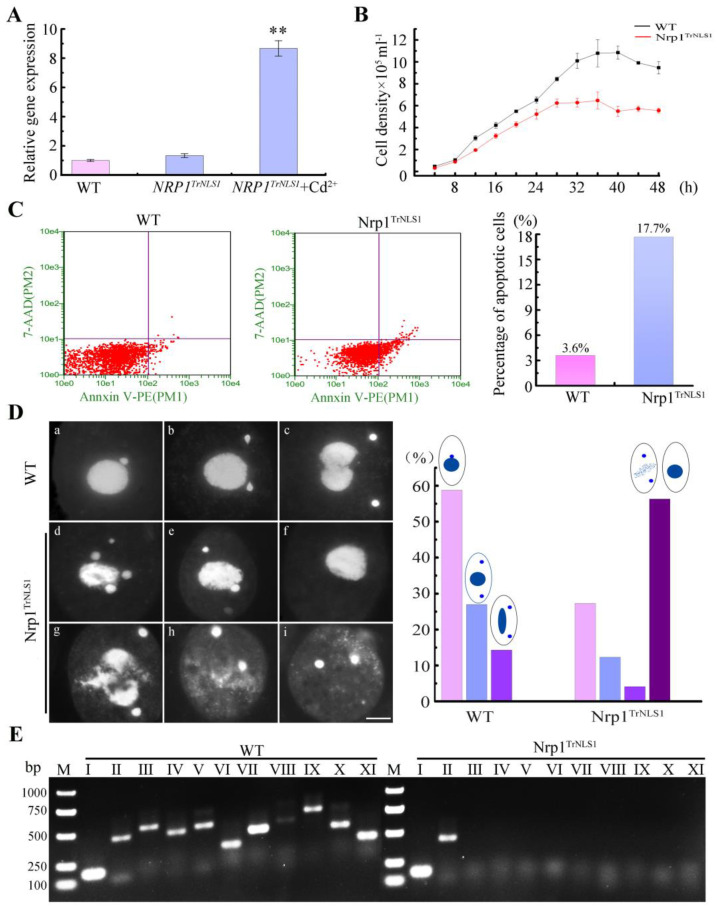
Proliferation and nuclear development of Nrp1^TrNLS1^ mutants. (**A**) Relative expression level of *NRP1*^TrNLS1^. Total RNA was isolated from cells in the vegetative growth stage. The relative expression level of *NRP1* was identified by qRT-PCR. Data were analysed statistically by independent samples t-test, asterisk indicate significant difference (** *p* < 0.01). (**B**) Proliferation of the Nrp1^TrNLS1^ mutant and WT cells; data represented three independent experiments. The cell concentrations were analyzed by paired-samples t-test, indicated the significant difference (*p* = 0.02 < 0.05) between Nrp1^TrNLS1^ mutant and WT cells. (**C**) Analysis of cellular apoptosis by flow cytometry. (**D**) Representative images of the division of MIC and MAC. (**a**–**c**) WT as the negative control, DNA was stained with DAPI. (**d**–**i**) Lost MIC and abnormally divided MAC were observed in the Nrp1^TrNLS1^ mutants. Scale bar, 10 µm. (**E**) M, trans 2 K plus DNA marker. MIC, specific sequences were amplified by PCR with 10 sets of primers. Primers Ⅰ was designed for JMJ1, primers Ⅱ–Ⅺ were designed for five different chromosomes in MIC. JMJ1 was used as the internal control.

The expression levels of HA-Nrp1, HA-Nrp1^TrNLS1^, HA-Nrp1^TrNLS2^, and HA-Nrp1^TrC^ under the *MTT1* promoter were upregulated by 12.3-, 8.7-, 2.3-, and 2.4-fold, respectively (Figure 2A and Supplementary Figure S2A). The overexpression of Nrp1 had no significant effect on *Tetrahymena* proliferation. However, the overexpression of Nrp1^TrNLS2^ and Nrp1^TrC^ mutant cells inhibited cellular proliferation, furthermore, the proliferation of the Nrp1^TrNLS1^ mutants was significantly inhibited when Nrp1^TrNLS1^ was overexpressed (Figure 2B and Supplementary Figure S2B). The Nrp1^TrNLS1^ mutant density was 59.7% for the wild type after 48 h culture. Furthermore, ~17.7% apoptotic cells formed in the Nrp1^TrNLS1^ mutant (Figure 2C), and 11.0% and 20.0% of the Nrp1^TrNLS1^ mutant cells showed MAC extrusion bodies and MIC loss after the cells were induced by Cd^2+^ for 24 and 48 h, respectively (Supplementary Figure S2C). Approximately 31.7% of the mutant cells showed disruption of MIC mitosis and loss of MICs after the cells were induced by Cd^2+^ for 96 h (Figure 2D(d–f)). A total of 24.3% of the Nrp1^TrNLS1^ cells showed abnormal MAC amitosis, MAC collapse, and degradation (Figure 2D(g,h)). The Nrp1^TrNLS2^ and Nrp1^TrC^ mutant cells revealed normal characteristics even after the cells were induced by Cd^2+^ for 96 h (Supplementary Figure S2D). The MAC chromosomes were generated by cleavage at chromosome breakage sequences (CBSs) consecutively spaced along the chromosome during sexual development. Specific primers can be used to amplify MIC-specific sequences [33]. The integrity of five chromosomes was analyzed in Nrp1^TrNLS1^ mutant cells. The left arm of the Ⅲ–Ⅴ chromosome and the right arm of the Ⅰ–Ⅴ chromosome were lost (Figure 2E). These results show that Nrp1^TrNLS1^ overexpression disturbs chromatin stability, promotes cellular apoptosis, and inhibits cellular proliferation in *Tetrahymena*. 

### 3.3. Overexpression of Nrp1^TrNLS1^ Abolishes Histone H3 Nuclear Import

**Figure 3:**
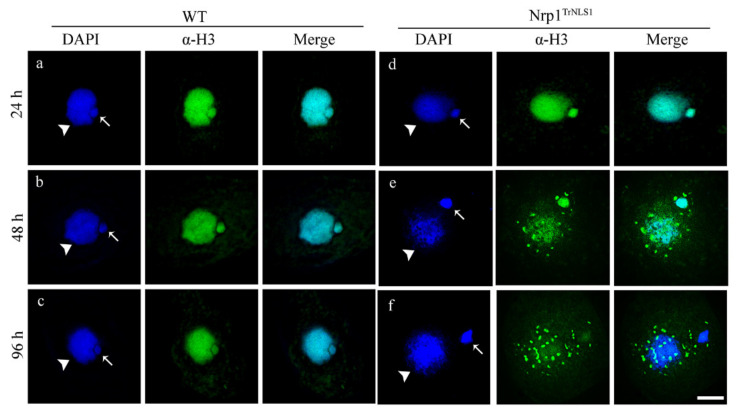
Overexpression of Nrp1^TrNLS1^ affects H3 nuclear import. Indirect immunofluorescence localization of H3. The WT (**a**–**c**) and Nrp1^TrNLS1^ (**d**–**f**) mutant cells were induced with 0.3 µg/mL Cd^2+^ for 24, 48, and 96 h. The primary antibody was histone H3 rabbit polyclonal antibody, and the secondary antibody was FITC-conjugated goat anti-rabbit. Arrows indicate MIC, arrowheads indicate MAC. Scale bar, 10 µm.

The nuclear import of histones is required for nucleosome assembly and chromosome formation. Histones H3 and H4 are transported by an evolutionarily conserved pathway and mediated by several proteins, including heat-shock protein HSP90, NASP, Asf1, and Importinβ, in yeast and human cells [10,34]. No significant difference in H3 fluorescent signals between Nrp1^TrNLS1^ and WT cells was detected after the cells were induced by Cd^2+^ for 24 h (Supplementary Figure S3). However, H3 signals decreased in the nucleus and H3 formed abnormal speckled signal in the cytoplasm after the Nrp1^TrNLS1^ cells were induced by Cd^2+^ for 48 h. Furthermore, the H3 signal disappeared in the nucleus, and speckle signals formed in the cytoplasm after Nrp1^TrNLS1^ cells were induced by Cd^2+^ for 96 h (Figure 3). These results show that Nrp1 mutation impairs the nuclear import of histone H3 and disturbs chromatin stability.

### 3.4. Modification of H3K56 Acetylation Decreases in Nrp1 Mutants

**Figure 4:**
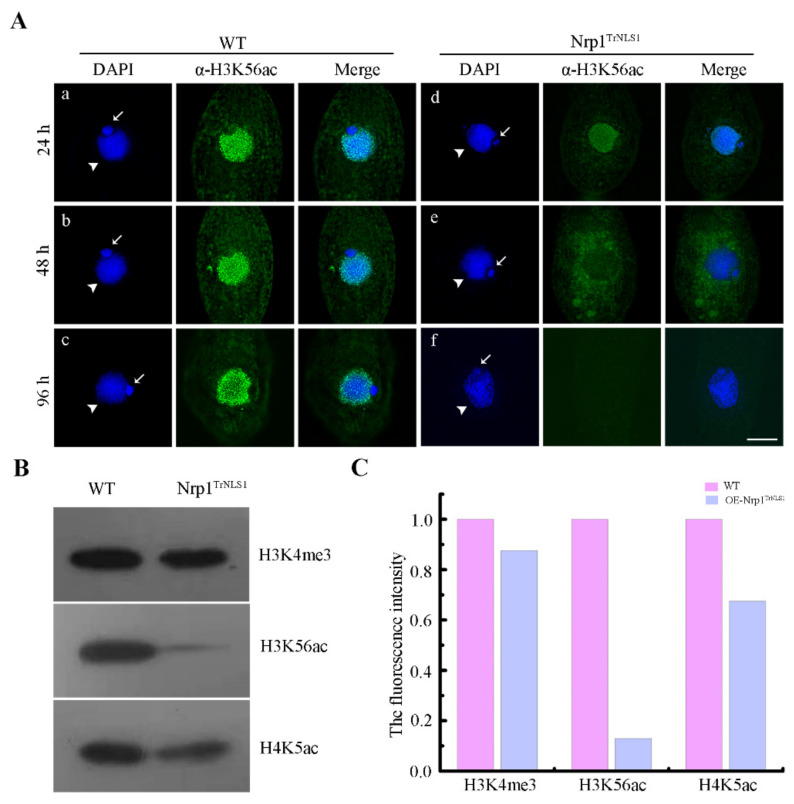
Nrp1 mutation leads to reductions in H3K56ac and H4K5ac. (**A**) Indirect immunofluorescence localization of H3K56ac and H4K5ac. The cells were induced with 0.3 µg/mL Cd^2+^ for 24, 48, and 96 h. The primary antibody was histone H3K56ac or H4K5ac rabbit polyclonal antibody, and the secondary antibody was FITC-conjugated goat anti-rabbit. White arrows indicate MIC, while white arrowheads indicate MAC. Scale bar, 10 µm. (**B**) Western blot analysis of H3K4me3, H3K56ac, and H4K5ac. Histone extracts prepared from WT and Nrp1^TrNLS1^ cells at the vegetative growth stage were induced with 0.3 µg/mL Cd^2+^ for 96 h. The extracted histone samples were separated by 15% SDS–PAGE. The gels were transferred to polyvinylidene difluoride membranes and probed by using anti-H3K4me3, H3K56ac, and H4K5ac antibodies. (**C**) The intensities of H3K4me3, H3K56ac, and H4K5ac were obtained by ImageJ software. The fluorescence of H3K4me3, H3K56ac, and H4K5ac in WT cells was arbitrarily set as 1, and the Nrp1^TrNLS1^ mutation fluorescence was normalized to signal from WT cells.

H3K56 acetylation appears to be widely distributed throughout polytene chromosomes in *Drosophila* [35]. H3K56 acetylation occurs extensively in MAC of *Tetrahymena* [36]. Asf1 and Rtt109 are required for global H3K56 acetylation in *Schizosaccharomyces pombe* [37]. However, Asf1 knockdown showed no effect on the acetylation level of H3K56 in *Tetrahymena* [26]. H3K56 acetylation levels significantly decreased when Nrp1^TrNLS1^ was expressed under Cd^2+^ induction for 24 h (Figure 4A(a,d)). The H3K56ac signal in the nucleus decreased, and irregular plaque signals were diffusely distributed in the cytoplasm after Nrp1^TrNLS1^ was expressed for 48 h (Figure 4A(b,e)); moreover, the H3K56ac signal was lost in the cells after Nrp1^TrNLS1^ was expressed for 96 h (Figure 4A(c,f)). The Western blotting analysis showed that the acetylation level of H3K56 was downregulated by 6.75-fold, that of H4K5 was downregulated by 0.48-fold, and the trimethylation level of H3K4 was downregulated by 0.1-fold after Nrp1^TrNLS1^ was expressed under Cd^2+^ induction for 96 h (Figure 4B,C), with total proteins of WT and Nrp1^TrNLS1^ as a control (Supplementary Figure S4A). However, the H3K56ac signal showed no significant difference among WT, Nrp1^TrNLS2^, and Nrp1^TrC^ mutant cells (Supplementary Figure S4B). These results strongly indicate that the overexpression of Nrp1^TrNLS1^ affects the acetylation modification of H3K56.

### 3.5. Nrp1^TrNLS1^ Mutants Affect the MIC Structure during Sexual Development

**Figure 5:**
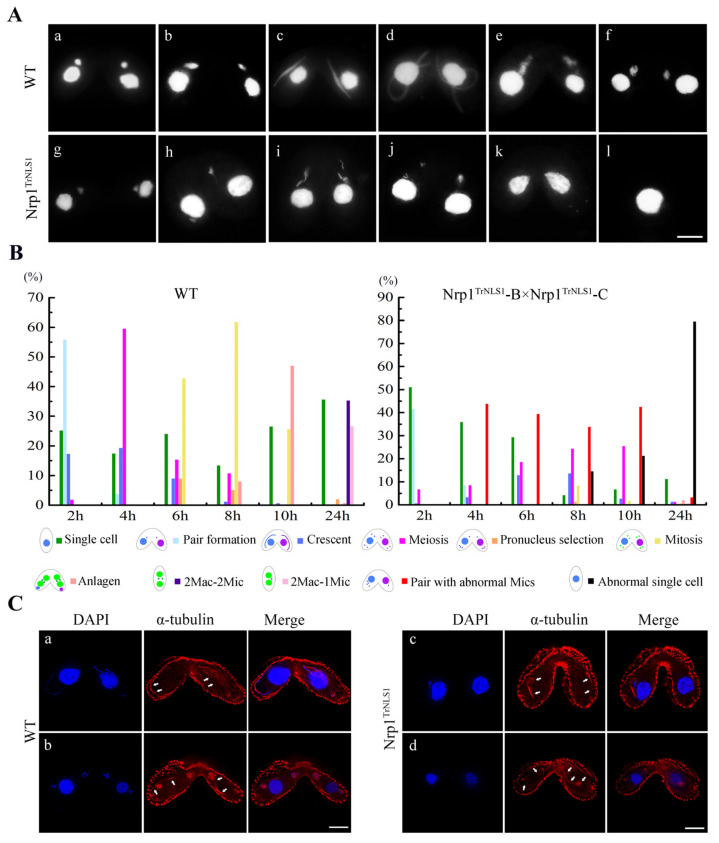
Micronuclear bouquet formation is affected in the Nrp1^TrNLS1^ mutant. (**A**) Microscopic analysis of changes in the MIC structure during meiosis. In the Nrp1^TrNLS1^ mutant, MICs failed to develop to the crescent stage. DNA was stained with DAPI; scale bar, 10 µm. (**B**) Development of the nucleus during sexual development. Percentage of different developmental stage cells during the sexual reproduction stage in the Nrp1^TrNLS1^ mutant and wild-type cell (n > 300). Cells were fixed at 2, 4, 6, 8, 10, and 24 h after mixing and staining with DAPI. (**C**) Spindle microtubule assembly was analyzed by indirect immunofluorescence localization. White arrowheads indicate spindle microtubules. The experiments were repeated thrice. (**a,b**) WT, (**c,d**) Nrp1^TrNLS1^ mutant. Scale bar, 10 µm.

*Tetrahymena* undergoes a remarkable MIC structural change during meiosis [38,39]. Meiosis I is divided into six stages, including stage I, spherical; stage II, egg shaped-spindle shape; stage III, torch shape; stage IV, thread-like crescent; stage V, drumstick shape; and stage VI, progressive shortening of MIC (Figure 5A(a–f)). We previously found that Nrp1 is strongly localized in meiotic MICs [27]. In this work, truncated Nrp1^TrNLS1^ was expressed under 0.3 μg/mL Cd^2+^ induction for 4 h to explore the function of Nrp1 during sexual development. In the mating mutants, dynamic MIC elongation was inhibited at prophase II or III (Figure 5A(g–j)). MICs failed to form a bouquet structure. Last, the abnormal Mics degraded and the mating pairs separated (Figure 5A(k,l)). At 6 h after mixing, 29.3% of the cells revealed abnormal MICs (Figure 5B). At 24 h after mixing, abnormal single cells accounted for over 80.0% of the cells found, meaning that Nrp1^TrNLS1^ inhibits MIC meiosis. MIC elongation is propagated by the polymerization of intranuclear microtubules during the early meiotic stage [39]. Nrp1 and the spindle apparatus co-localize [27]. The nuclear envelope and intranuclear microtubules are still able to elongate in the absence of Cna1p [30]. In WT cells, microtubules were distributed around the MIC periphery and formed a spindle structure (Figure 5C(a,b)). In contrast, the intranuclear and perinuclear microtubule bundles failed to elongate in Nrp1^TrNLS1^ mutant cells (Figure 5C(c,d)). 

### 3.6. Nrp1^TrNLS1^ Mutant Affects Macronuclear Genome Transcription

**Figure 6:**
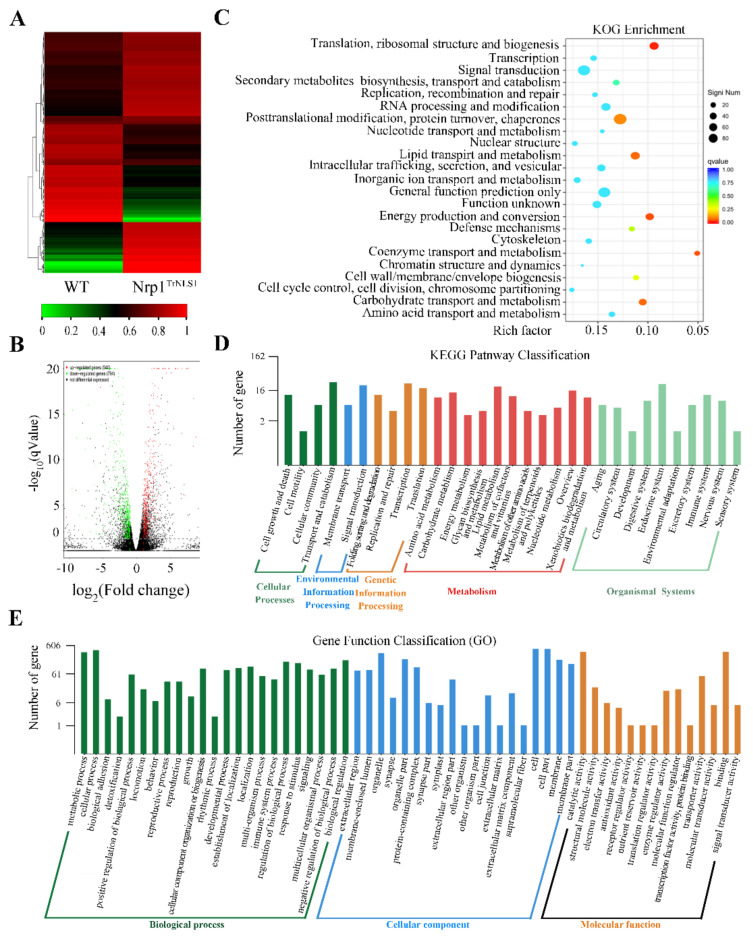
Identification of differentially expressed genes in Nrp1^TrNLS1^ mutant. (**A**) Heatmap of DEGs in WT and Nrp1^TrNLS1^ cells. DEGs with a |log2FC| > 1 are indicated in red, and DEGs with a |log2FC| < 1 are indicated in green. (**B**) Volcano plots of DEGs between samples. The threshold q < 0.05 was used to determine the significance of DEGs. Red and green dots represent up- and downregulated genes, respectively, and black dots indicate transcripts that did not change significantly in WT and Nrp1^TrNLS1^ cells. (**C**) KOG enrichment analysis of Nrp1^TrNLS1^ and WT cells. (**D**) KEGG pathway-enrichment analysis of Nrp1^TrNLS1^ and WT cells. (**E**) GO-term enrichment analysis of WT and Nrp1^TrNLS1^ cells.

H3K56 acetylation is associated with actively transcribed genes in *S. cerevisiae* [14]. RNA-Seq analysis was performed on the Nrp1^TrNLS1^ mutant, and DEGs were hierarchically clustered to obtain a differential gene expression in Nrp1^TrNLS1^ cells and explore whether the Nrp1^TrNLS1^ mutant affects genome transcription in *Tetrahymena*. All RNA-Seq data are available in the NCBI Sequence Read Archive Database (https://dataview.ncbi.nlm.nih.gov/object/, BioProject, PRJNA795692, accessed on 10 January 2022). The heatmap and volcano plot revealed that 794 DEGs are downregulated, whereas 540 DEGs are upregulated (Figure 6A,B). The functional groups were divided into 23 groups (Figure 6C). The annotated transcripts were mapped to the KEGG pathway, and the functional groups were divided into five categories, namely cellular, environmental, genetic, metabolism, and organismal systems. A total of 1334 DEGs were mapped to 31 KEGG pathways. The DEGs were enriched in cell growth and death, transport and catabolism, and transcription (Figure 6D). The 1334 DEGs were classified as 56 functional groups to obtain the functional annotations of the DEGs for Nrp1^TrNLS1^ overexpression. Our results indicated that the DEGs are evidently enriched in chromatin structure and dynamics, signal transduction mechanisms, post-translational modification, and chaperones (Figure 6E).

**Figure 7:**
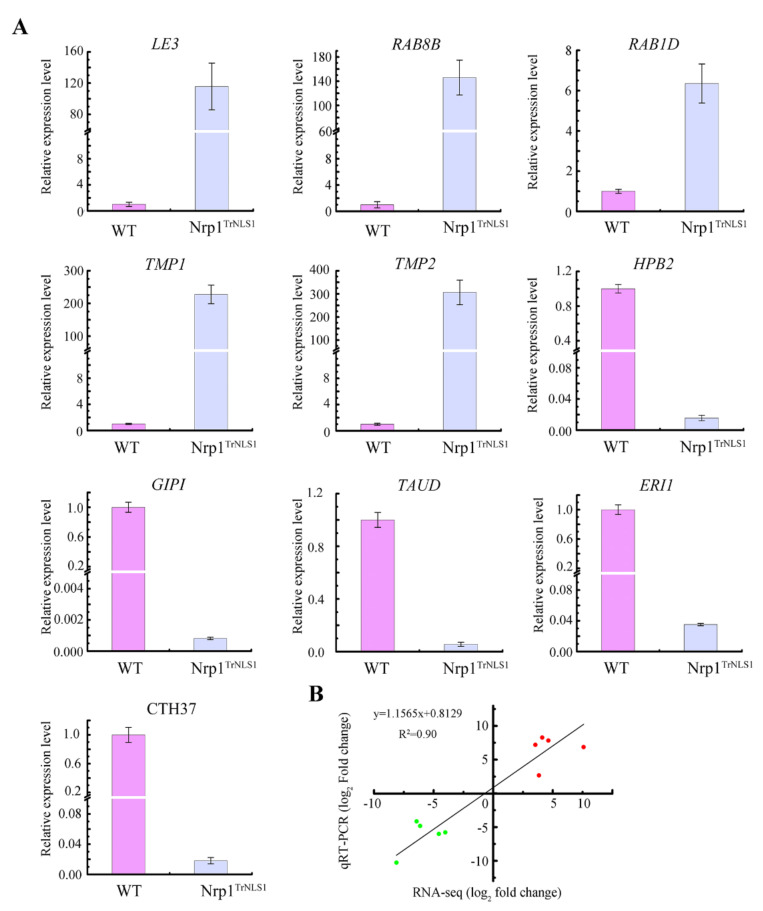
Relative expression level of 10 differentially expressed genes. (**A**) The expression level of 10 genes was determined by qRT-PCR. (**B**) Comparison of the log2 of gene expression ratios obtained between the RNA-Seq data and qRT-PCR. The qPCR log_2_ value of the expression ratio (*y*-axis) was plotted against the value from the RNA-Seq (*x*-axis). R^2^ represents the correlation coefficient between qRT-PCR and RNA-Seq.

Ten DEGs were evaluated through qRT-PCR to validate the accuracy and reliability of transcriptome sequencing. The expression levels of five genes, namely *LE3*, *RAB8B*, *RAB1D*, *TMP1*, and *TMP2*, were upregulated. By contrast, the expression levels of the genes *HPB2*, *GIPI*, *TAUD*, *ERI1,* and *CTH37* were downregulated (Figure 7A). Pearson correlation coefficients was calculated by SPSS to assess the correlation between different platforms. Overall, the qRT-PCR measurements were correlated with the RNA-Seq results (Pearson coefficient R^2^ = 0.90). These results suggest that RNA-Seq is a reliable tool for finding DEGs in Nrp1 mutant cells (Figure 7B).

## 4. Discussion

### 4.1. NLS1 or NLS2 Is Sufficient for Directing Nrp1 Nuclear Import

NASP is highly conserved throughout eukaryotes and critical for the proper growth and development of complex eukaryotic organisms [32,40,41]. Human NASP contains the typical TPR and C-terminus domains, and these distinct structural domains interact with linker and core histones [42]. Human NASP occurs in two major forms, namely tNASP, which is found in gametes, embryonic cells, and transformed cells; and sNASP, which is found in all rapidly dividing somatic cells. The C-terminal fragment of sNASP is unable to bind to any of these histones [43]. However, the NH2-terminal fragment of sNASP protein interacts with a discrete peptide epitope located within the globular domain of histone H3 [40]. The C-terminal domain of tNASP contains only one NLS, and the NLS deletion mutant is retained in the cytoplasm [44]. The C-terminus of Hif1 (human homolog of NASP) is essential for its proper nuclear localization in *S. cerevisiae* [45]. However, we found that the C-terminal domain of Nrp1 contains two nuclear localization signals, NLS1 and NLS2 (Figure 1C). The truncated NLS1 and NLS2 mutant HA-Nrp1^TrC^ are retained in the cytoplasm and enriched at the periphery of the nucleus. However, HA-Nrp1^TrNLS1^ lacking NLS1 and HA-Nrp1^TrNLS2^ lacking NLS2 could still be located in the MIC and MAC in *Tetrahymena* (Figure 2B). The single nuclear localization signal NLS1 or NLS2 is sufficient for directing Nrp1 nuclear import. 

### 4.2. Nrp1 Mutation Leads to Nuclear Degradation and Cellular Apoptosis

The assembly and deposition of histones into nucleosomes represent a major challenge in ensuring genomic integrity and regulating genomic processes [40]. Eukaryotes have evolved a precise and highly tuned histone supply system, and histone pools respond quickly when there is a histone turnover and active replication of genome [46,47]. Overexpression of human tNASP clearly affects the progression of cells through the cell cycle [48]. In *Tetrahymena*, the overexpression of Nrp1 has less effect for cellular proliferation, but the overexpression of Nrp1^TrNLS1^ significantly inhibits cellular proliferation. MAC is critical for *Tetrahymena* survival and proliferation during the vegetative growth stage. As the number of generations of cell division increases, nuclear division defects became more serious in the Nrp1^TrNLS1^ mutant. Furthermore, mitosis of MIC mitosis was also abnormal, and the MAC disintegrated and dispersed in the cytoplasm. In contrast, HA-Nrp1^TrC^ and HA-Nrp1^TrNLS2^ showed no abnormal phenotype. The Nrp1^TrNLS1^ mutant clearly affected Nrp1 function.

NASP depletion induces the apoptosis of PC-3 and HeLa cells [49]. NASP knockdown activates anti-apoptotic factor *BACH2* and *RunX1T1*, which enhances apoptosis in SMMC cells [50]. In mammals, NASP is an essential protein in mammals that functions in histone transport pathways and the maintenance of a soluble reservoir of histones H3/H4 [51]. HSP90, Asf1, and Importinβ are also highly conserved components in the histone H3/H4 transport pathway [52,53]. Asf1-Impβ6-Nrp1 controls the nuclear transport of histones H3 and H4 in *Tetrahymena* cells [26,54]. Newly synthesized histones cannot be directly transported and assembled on chromatin, and posttranslational modification is critical for the transport and assembly of histones. Acetylation of the newly synthesized H3 is a transient modification. Once these histones are transported into the nucleus and assembled into chromatin, they are deacetylated during chromatin maturation [55]. H3K56 functions as a point of contact with DNA at the entry/exit point of the nucleosome, and acetylation may be capable of physically altering the contact between histone H3 and DNA [56]. Defects in H3K56 acetylation result in sensitivity to genotoxic agents that causes strand breaks during replication. Nevertheless, constitutive H3K56 acetylation also results in poor growth, spontaneous DNA damage, and chromosome loss, thus suggesting that abnormally modified H3K56 disturbs the chromosome structure [57,58]. In *Tetrahymena*, H3K56ac is specifically located in MAC [59]. However, a clear ortholog of either Rtt109 or p300/CBP is lacking, and Asf1 knockdown does not affect the acetylation level of H3K56 [26]. Nrp1^TrNLS1^ overexpression severely affected the acetylation level of H3K56, and newly synthesized H3 was not transported into the nucleus. Overexpression of Nrp1^TrNLS1^ disturbs the normal H3 nuclear import signaling pathway. 

### 4.3. Nrp1 Mutations Affect Genomic Transcription

Transcriptional regulation is a central molecular event in gene expression associated with nearly every aspect of biological activity. Elaborate modulations of chromatin structure, including the PTMs of histones, are key events associated with transcriptional regulation [60]. Methylation of H3K4 is intimately related to active transcription [61], and H3K9me3 and H3K27me3 promote heterochromatin formation and gene silencing [62]. H3K56ac is a potential epigenetic marker of gene transcription and occurs within actively transcribed genes [63,64]. Nrp1^TrNLS1^ overexpression severely affects the acetylation level of H3K56. Decreased acetylation of H3K56 could contribute to enhancements in the transcription barrier. RNA-Seq analysis showed that 794 DEGs are downregulated, whereas 540 DEGs are upregulated, in the Nrp1^TrNLS1^ mutant (Figure 6A,B). H3K56 acetylation correlates positively with the binding of Nanog, Sox2, and Oct4 transcription factors, which perturbs H3K56 acetylation, and decreases Oct4–H3 binding in human ES cells [65]. In this work, we failed to identify the corresponding homologs in *Tetrahymena*. However, GO terms and KEGG pathway enrichment analysis indicated that several DEGs are involved in the activity of transcriptional factors in Nrp1^TrNLS1^ cells (Figure 6D,E), including five transcription factors (i.e., *TFⅡB*, *TFⅡD*, *TFSMA*, *RRM43*, and *ZFC2H2*), two histone deacetylases (i.e., *THD10* and *TFD16*), and one histone acetylase (i.e., *ELP3*) (Supplementary Table S3). *TFIIB* plays an essential role in the preinitiation complex assembly and initiation of transcription by recruiting RNA polymerase II to the corresponding promoter [66]. *TFIID* enables RNA polymerase II promoter-proximal pausing [67]. Chaperone-dependent nucleosome unwrapping is essential for gene transcription. Histone acetyltransferase *ELP3* was downregulated by 2.25-fold and histone deacetylases *THD10* and *TFD16* were downregulated by 4.7- and 2.8-fold, respectively (Supplementary Table S3). These results indicate the existence of a dynamic balance between histone acetylation and deacetylation. Taken together, the results show that the histone chaperone Nrp1 regulates H3 transport and H3K56 acetylation and affects chromatin stability and genome transcription in *Tetrahymena*.

## Data Availability

Not applicable.

## Supplementary Materials

The following are available online at https://www.mdpi.com/article/10.3390/cells11030408/s1. Figure S1: Bioinformatic analysis of *NRP1* and predicted secondary structure, identification of Nrp1 mutant cell lines, and Western blotting analysis of HA-Nrp1. Figure S2: Identify the expression level of *NRP1* in each mutant cells by qRT-PCR, analysis of cell growth rate of wild type and three mutant strains, statistical analysis of micronuclear mitosis, and macronuclear expelled body in Nrp1 mutant cells. Figure S3: Fluorescence signal intensity analysis of H3. Figure S4: Identification of Nrp1 mutant cell lines and indirect immunofluorescence analysis of H3K56ac in different Nrp1 mutant cells. Table S1: Primers used in this work. Table S2: Summary statistics of transcriptome sequencing. Table S3: Transcription related factor genes response to Nrp1 mutation.
