# Supplementary material for: Histone Chaperone Nrp1 Mutation Affects the Acetylation of H3K56 in Tetrahymena thermophila
Source: Cells. 2022 Jan 25;11(3):408. doi: 10.3390/cells11030408 (PMC8833950; doi:10.3390/cells11030408)
Supplement: Supplementary file 1 [file cells-11-00408-s001.zip › cells-1499236-supplementary.pdf]

Histone chaperone Nrp1 regulates acetylation of H3K56 and transcription of genome in *Tetrahymena thermophila*

Yinjie Lian [1], Huijuan Hao[1], Jing Xu[1,2], Tao Bo [1] and Wei Wang [1,*]

Supplemental Information:
Supplemental Figures S1–S4
Supplemental Tables S1–S3

Supplementary Figure S1

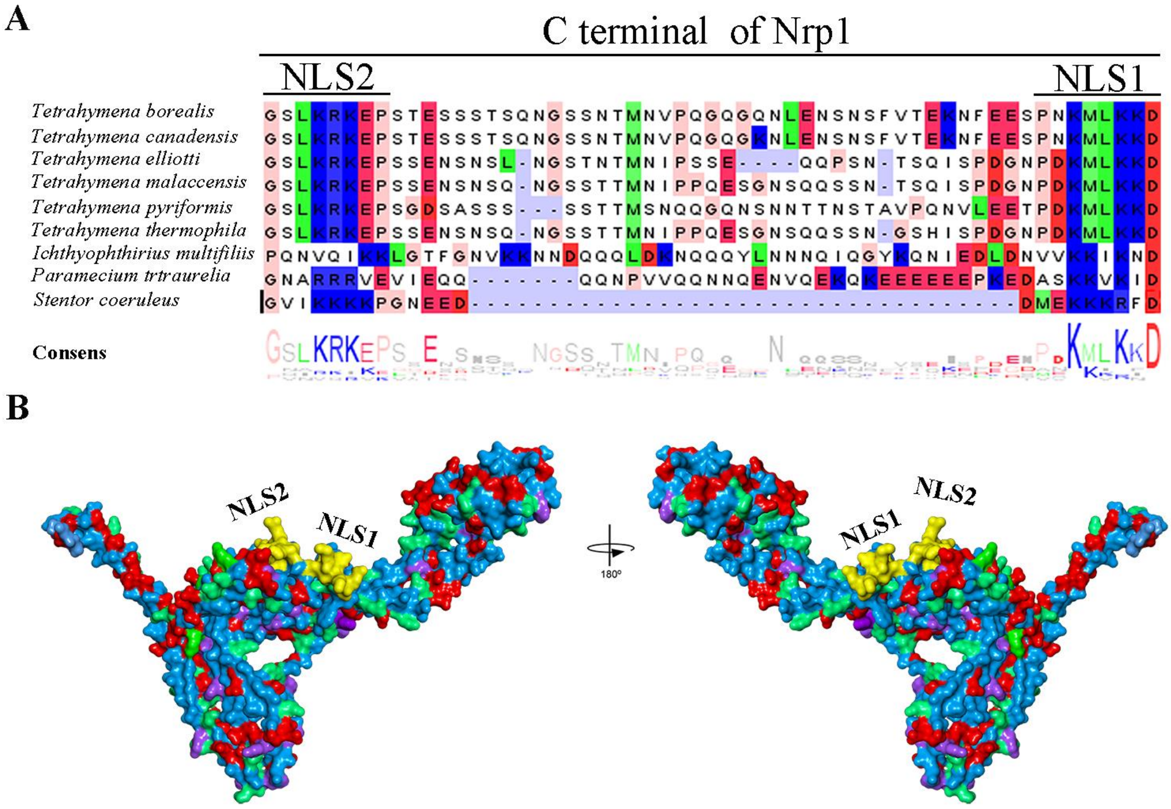

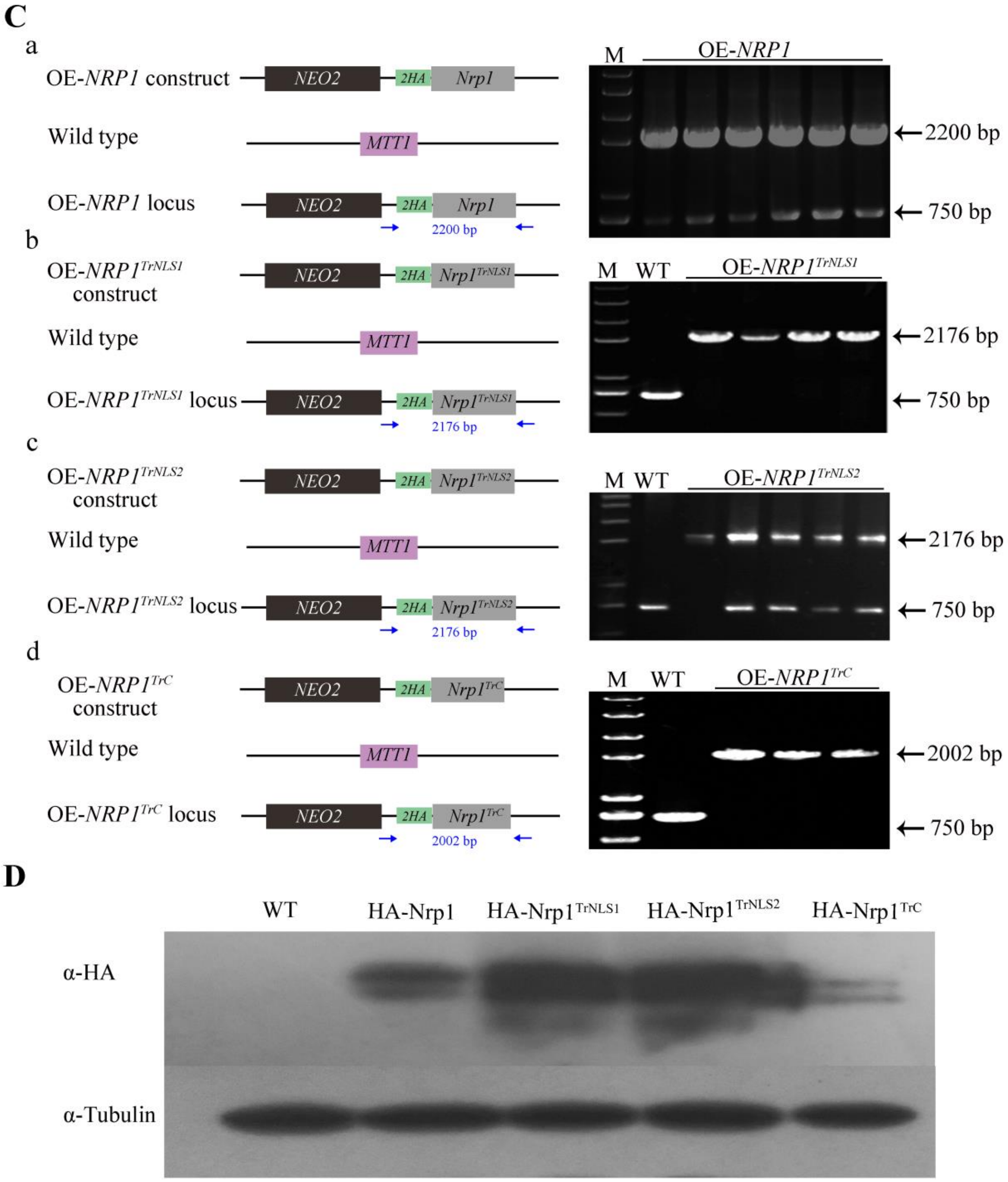

Supplemental Figure S1. (**A**) Bioinformatic analysis of NRP1 from *T.thermophila*. Cluster analysis of the C-terminal domain of Nrp1 protein from 9 different free-living ciliates. (**B**) Predicted secondary structure of Nrp1 by Phyre2, red represents acidic amino acids, green represents basic amino acids, purple represents aromatic amino acids, yellow indicates nuclear localization signal. (**C**) Identification of Nrp1 mutant cell lines. Schematic representation of recombinant HA-Nrp1, HA-Nrp1^{TrNLS1}, HA-Nrp1^{TrNLS2}, and HA-Nrp1^{TrC} mutants in *T. thermophila*. The identification of Nrp1 mutants. WT and mutant loci were confirmed by PCR using the primers OE-J-*NRP1*-F/OE-J-*NRP1*-R (Table S1). Arrows indicate WT and mutated locus. (**D**) Western blotting analysis of HA-Nrp1 in mutant cells. After cells were induced by Cd^{2+} for 12 h, 1×10^7 cells were collected during meiotic stage. The whole cell extracts (WCE) of WT, HA-Nrp1, HA-Nrp1^{TrNLS1}, HA-Nrp1^{TrNLS2}, and HA-Nrp1^{TrC} mutant were prepared. Top panel was probed with anti-HA antibody, the bottom panel was probed with anti-tubulin antibody as a loading control.

Supplementary Figure S2

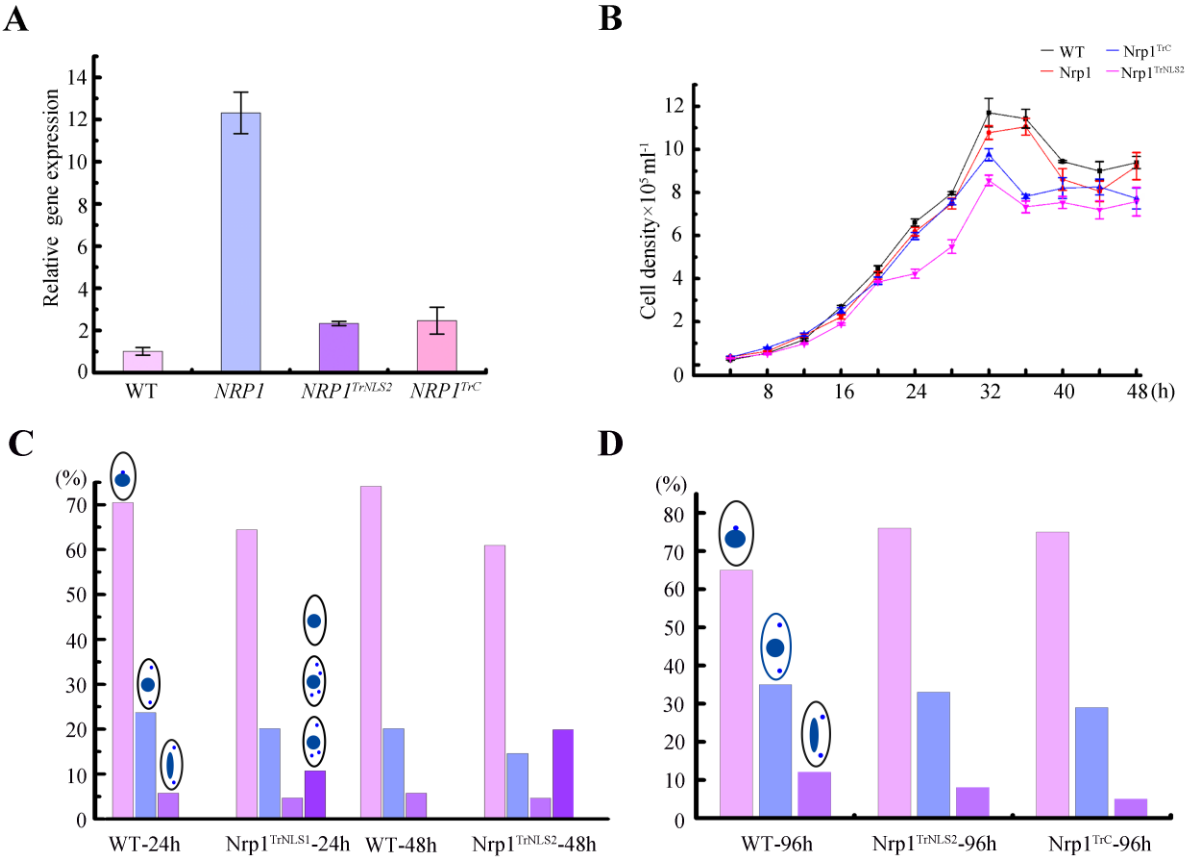

Supplemental Figure S2. (**A**) Relative expression level of *NRP1* and *NRP1* mutants. Total RNA was extracted from WT, *Nrp1*, $Nrp1^{TrNLS2}$ and $Nrp1^{TrC}$mutant cells. qRT-PCR was performed with the RT-*NRP1*-F/RT-NRP1-R and 17s-F/17s-R primer pairs (Table S1) using an SYBR Green II mix (SYBR®Premix Ex Taq™ Kit, Takara). The steps are as follows: heat for 30 s at 95 °C, followed by 40 cycles of 5 s at 95 °C and an extension for 30 s at 60 °C. The relative quantifications of the *NRP1* mRNA expression levels were normalized by using 17S rRNA as an internal control. (**B**) Proliferation of wild type and three mutant strains. The growth curve of WT, HA-Nrp1, HA-$Nrp1^{TrNLS2}$, and HA-$Nrp1^{TrC}$ cells under 0.3 ug/mL Cd^{2+} induction. Initial cell concentration was 0.15 × 10^5 cells/ml. Data were the mean ± standard deviation of three independent experiments. The cell concentrations (36 h, 40 h and 44 h) were analyzed by paired-samples T-test, the cell concentration has no significant change between overexpression of Nrp1 and WT cells ($P = 0.165 > 0.05$); the cell concentration is different between $Nrp1^{TrNLS2}$ and WT ($P = 0.029 < 0.05$) or between $Nrp1^{TrC}$ and WT cells ($P = 0.040 < 0.05$). (**C**) The analysis of macronucleus extruded body in Nrp1 mutant cells. After cells were induced with 0.3 μg/mL Cd^{2+} for 24h and 48h, percentage of macronucleus extruded body, micronuclear mitosis, and macronuclear amitosis in WT and $Nrp1^{TrNLS1}$ mutants (n = 300). Magenta represents the interphase, blue represents micronuclear mitosis, purple represents macronuclear amitosis, dark purple represents abnormal single cells. (**D**) The analysis of macronucleus extruded body in Nrp1 mutant cells after cells were induced with 0.3 μg/mL Cd^{2+} for 96h. Percentage of micronuclear mitosis and macronuclear amitosis in WT, $Nrp1^{TrNLS2}$, and $Nrp1^{TrC}$ (n = 300). Magenta represents the interphase, blue represents micronuclear mitosis, purple represents macronuclear amitosis.

Supplementary Figure S3

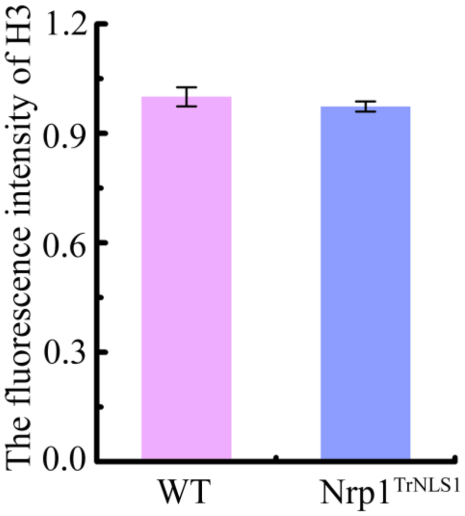

Supplemental Figure S3. Fluorescence signal intensity analysis of H3. Fluorescence intensity of H3 in overexpression of Nrp1^{TrNLS1} and WT cells. Fluorescence intensity data obtained through ImageJ V1.8.0, data were the mean ± standard deviation of three independent experiments. The fluorescence of H3 in WT cells was arbitrarily set as 1, and the fluorescence of H3 in overexpression of Nrp1^{TrNLS1} cells was normalized.

Supplementary Figure S4

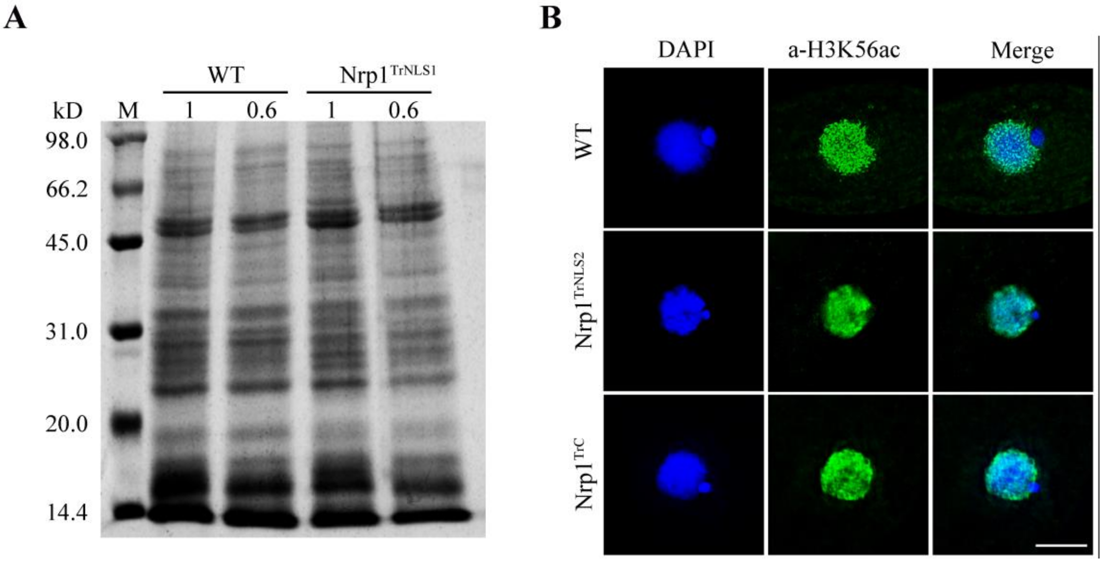

Supplemental Figure S4. (**A**) SDS-PAGE analysis of total proteins. Whole cell extracts were prepared from WT and Nrp1^{TrNLS1} after cells were induced with 0.3 μg/mL Cd^{2+} for 96 h from vegetative growth stage. 12 % SDS-PAGE analysis of WT and Nrp1^{TrNLS1}. M: protein molecular weight markers; lane 1–2: whole cell extracts were prepared from WT; lane 3–4: whole cell extracts were prepared from Nrp1^{TrNLS1}. (**B**) Indirect immunofluorescence analysis of H3K56ac in different Nrp1 mutant cells. Indirect immunofluorescence analysis of WT, Nrp1^{TrNLS2}, and Nrp1^{TrC} cells during vegetative growth stage using H3K56ac antibody. White arrows indicate the micronuclei; white arrowheads indicate the macronuclei. Scale bar, 10 μm.

Supplementary Table S1: Primers used in this work.

PCR primers	Sequence
OE-*NRP1*-F	GGATCCATGAGTTCTGACAATAGTGAATAAG
OE-*NRP1*-R	GGCGCGCCTCAATCCTTTTTGAGCATTTTATCTG
OE-J-*NRP1*-F	GCTACGTGATTCACGATTTATGCAATG
OE-J-*NRP1*-R	CGAAACTGATTTTATGCAATTATGAATTAC
OE-*NRP1*^{TrNLS1}-F	GGATCCATGAGTTCTGACAATAGTGAATAAG
OE-*NRP1*^{TrNLS1}-R	GGCGCGCCGTTTCCATCAGGACTAATATGAG
OE-*NRP1*^{TrNLS2}-F1	GGATCCATGAGTTCTGACAATAGTGAATAAG
OE-*NRP1*^{TrNLS2}-R1	GCTGTTTTCAGAAGAAAATGTACCTAAATTTTTGATAGGAAC
OE-*NRP1*^{TrNLS2}-F2	TTTAGGTACATTTTCTTCTGAAAACAGCAATTCTTAAAAT
OE-*NRP1*^{TrNLS2}-R2	GGCGCGCCTCAATCCTTTTTGAGCATTTTATCTG
OE-*NRP1*^{TrC}-F	GGATCCATGAGTTCTGACAATAGTGAATAAG
OE-*NRP1*^{TrC}-R	GGCGCGCCTCATTATGAAGGTTAGGCAAATTG
GFP-*NRP1*-F	CTCGAGCAATTTGCCTAACCTTCATAAAATAGTG
GFP-*NRP1*-R	GGTACCATCCTTTTTGAGCATTTTATCTGGGTT
RT-*LE3*-F	GCTTGTACTGATACTAATGCCACTG
RT-*LE3*-R	CGAGACAATTGCATGAGGTGACAAG
RT-*RAB8B*-F	CTACCTACAATTGGTATTGATTATGAG
RT-*RAB8B*-R	ATATGCAAGTATAATACCCATAGAGCC
RT-*RAB1D*-F	GCTATTACTGGAAGCTCATCAGTCG
RT-*RAB1D*-R	CAAATCTTTCTTAACCAGCTGTATCCC
RT-*TMP1*-F	CTCTTGCGATAGTTCTAATACTAGTTG
RT-*TMP1*-R	GTCTGTTTTGCATTTCACGTAGTAGC
RT-*TMP2*-F	CTCTGTTAATCTTACATGCAACAGTTC
RT-*TMP2*-R	TGGCTGATAGGTTGGTTTGCAATTG
RT-*HPB2*-F	AGTATATTGGAGATAGCCACAAGCCT
RT- *HPB2*-R	CACATTCGTAAGAGACAAGATTAAGTC
RT-*GIPI*-F	CAACTTGTTGATTGTGTCAGCCTAG
RT-*GIPI*-R	CTCAATTGCATATCTAACACCATCTTC
RT-*TAUD*-F	CTTATGGGAACACGGAACCATTTGC
RT-*TAUD*-R	AAGGAGGACGGACAATTATGGAACC
RT-*ERI1*-F	TCTGCATGCACTAACTTCCCTACTG
RT-*ERI1*-R	AGATTAACCATAGTCATTACTCCAAGC
RT-CTH37-F	AGAGCTTCCTGATACTTGGCTTTGG
RT-CTH37-R	GTATTATGGGATATGTTAGTTACGTCC
17s-F	CCTGGGAAGGTACGGGTAAT
17s-R	AAGGTTCACCAGACCATTCG
CBs1-left-F	CCAAAAAAAATTCATAAATAGACAGC
CBs1-left-R	ATCAAAGGGCTTTCTACTCATC
CBs1-right-F	ACCACAAAAGTAAGTTAAGTACG
CBs1-right-R	ATATAGCCATTGATTAGTGTGTG
CBs2-left-F	TGTCTTGATGAACATAGGCAC
CBs2-left-R	TATAGGTGAGTGACTTGCTAC
CBs2-right-F	TGTGTTTTTCATCAAAACATAC
CBs2-right-R	TTGGTTATGCTACAACTGTTG
CBs3-left-F	ATTTCAGCTTGTTGTCTGTTTGG
CBs3-left-R	TTTACCCAACTTGATCTCTCATCC
CBs3-right-F	TCAACCTCTAAAGCACTCATTAC
CBs3-right-R	AACTCTTGCCTAACCAAACATTC
CBs4-left-F	ACGAGTTTATTTGATGCTAATTAG
CBs4-left-R	ACAAATAGATAGCTTC

CBs4-right-F	TCTAACTAAGAGCTTATTCAGG
CBs4-right-R	TGCTTTTACACCAGCCAATCTAG
CBs5-left-F	AGTAATCATCTTGAAGAAGTTGG
CBs5-left-R	TCAGAGATGTGTTTCCTTGATGG
CBs5-right-F	TGTAATGAGAAAGTGTAAACTAGC
CBs5-right-R	AACCTTACTCTATGTTTCAACG
JmJ1-F	TTTGAGCTTTCAATTCCAAAAGG
JmJ1-R	GTTCTATTTAGTGCTGGGAATG

Underline indicates recognition sites of the restriction enzymes.

Supplementary Table S2 Summary statistics of transcriptome sequencing

Sample name	Total Bases Count	Total Reads Count	Q20	Q30	GC content
WT-1	6028819832	42538440	94.57%	85.20%	37.83%
WT-2	5527652040	38927376	94.78%	85.74%	38.13%
WT-3	5436951511	38256940	94.78%	85.71%	37.85%
Nrp1^{TrNLS1}-1	5319459665	37560090	94.68%	85.40%	37.50%
Nrp1^{TrNLS1}-2	6432303568	45188694	95.42%	87.29%	36.79%
Nrp1^{TrNLS1}-3	5625057286	39548670	94.77%	85.64%	36.14%

Supplementary Table S3 Transcription related factor genes response to Nrp1 mutation

Gene ID	Gene name	Log_2 Fold change
TTHERM_00418580	TFIIB	−1.02
TTHERM_000105239	TFIID	−1.38
TTHERM_00688320	TFSMA	−1.22
TTHERM_00683070	RRM43	−1.15
TTHERM_00698680	ZFC2H2	−2.08
TTHERM_00859330	THD10	−2.23
TTHERM_00194150	THD16	−1.48
TTHERM_00637650	ELP3	−1.16
